# A biomechanical model for the relation between bite force and mandibular opening angle in arthropods

**DOI:** 10.1098/rsos.221066

**Published:** 2023-02-15

**Authors:** Frederik Püffel, Richard Johnston, David Labonte

**Affiliations:** ^1^ Department of Bioengineering, Imperial College London, London SW7 2AZ, UK; ^2^ School of Engineering, Materials Research Centre, Swansea University, Swansea SA2 8PP, UK

**Keywords:** insect biomechanics, bite forces, mandible gape, muscle physiology

## Abstract

Bite forces play a key role in animal ecology: they affect mating behaviour, fighting success, and the ability to feed. Although feeding habits of arthropods have a significant ecological and economical impact, we lack fundamental knowledge on how the morphology and physiology of their bite apparatus controls bite performance, and its variation with mandible gape. To address this gap, we derived a biomechanical model that characterizes the relationship between bite force and mandibular opening angle from first principles. We validate this model by comparing its geometric predictions with morphological measurements on the muscoloskeletal bite apparatus of *Atta cephalotes* leaf-cutter ants, using computed tomography (CT) scans obtained at different mandible opening angles. We then demonstrate its deductive and inductive utility with three examplary use cases: Firstly, we extract the physiological properties of the leaf-cutter ant mandible closer muscle from *in vivo* bite force measurements. Secondly, we show that leaf-cutter ants are specialized to generate extraordinarily large bite forces, equivalent to about 2600 times their body weight. Thirdly, we discuss the relative importance of morphology and physiology in determining the magnitude and variation of bite force. We hope that a more detailed quantitative understanding of the link between morphology, physiology, and bite performance will facilitate future comparative studies on the insect bite apparatus, and help to advance our knowledge of the behaviour, ecology and evolution of arthropods.

## 1. Introduction

Bite forces are a performance metric paramount to animal behaviour, ecology and evolution [1]. They determine access to food sources [2–7], fighting ability and reproductive success [8–11], as well as the ability to escape predators [12]. Strikingly, the majority of bite force studies have been conducted on vertebrates and crustaceans [2–8,11,13–52]. In sharp contrast, comparatively few studies exist for the hyperdiverse non-crustacean arthropods (but see [9,10,12,53–67]).

This disparity is as striking as it is surprising, as arthropods rely on bite forces just as much as vertebrates: to fight over access to food and mating partners [9,10], and in other key behaviours such as nest building [68]. The arguably most important role of bite forces in arthropods, however, is that they determine the ability to mechanically process food [69–71]. Indeed, plant-eating insects have an enormous impact on our lives. They destroy around 11% of our crops, causing billions of US dollars in economic damage, may jeopardize global food security in a warming climate [72–76], and affect entire ecosystems [77]. To provide just two illustrative examples, leaf-cutter ants accelerate the cycling of nutrients in the Neotropics through massive defoliation and decomposition of plant material [78], and seed-harvesting ants increase the dispersion rate and regeneration of myrmecochorous plants [79]. The need to mechanically process plant material in order to feed is likely one of the driving factors in the evolutionary arms race between plants and insects, and thus constitutes a significant aspect of insect diversity [80–82].

Despite this broad significance, fundamental knowledge about bite performance across the arthropod tree of life is scarce. A reasonable and indeed common starting point to characterize bite performance within and between species may be the magnitude of the peak bite force [83]. This peak force, however, can be difficult to measure because arthropods are often small; it is also challenging to predict, as it depends on the geometry of the bite apparatus and the physiology of muscle, and both vary substantially across arthropods [23,82]. To make matters worse, bite force typically varies with the mandibular opening angle or gape, adding further complexity [4,7,10,26,38,44,48,56,60,84]. This variation is not merely a complication, but it is functionally relevant and may reflect species-specific demands. For example, masticating bats produce largest bite forces at small opening angles [26,48], whereas snail-eating carps produce maximum forces at comparatively large angles [4]; predatory king salmons produce maximum bite force at a relatively larger mandible gape than filter-feeding pink salmons [7]; and stag beetles generate largest bite forces at opening angles that are frequently used during combat [10]. The ecological and behavioural needs of each species hence likely determine what constitutes a functionally advantageous variation of bite force with mandibular opening angle, so leading to selection on the morphological and physiological properties of the musculoskeletal bite apparatus.

Although bite forces depend on a large number of anatomical and physiological parameters, they are of mechanical origin, which makes them amenable to exact analysis from first principles. A quantitative model which predicts the magnitude and variation of bite force with opening angle from morphology and physiology of the bite apparatus would provide a powerful tool to study comparative bite performance and head anatomy, and may thus increase our understanding of arthropod behaviour, ecology and evolution. In the following paragraphs, we derive such a model and then validate it using *A. cephalotes* leaf-cutter ants as the model organism. We demonstrate the utility of the model by extracting the force–length properties of the mandible closer muscle from *in vivo* bite force measurements, discuss the morphology and exceptional performance of the leaf-cutter ant bite apparatus in an ecological and comparative context and discuss the possibility and accuracy of minimal bite force models which allow colleagues to predict the magnitude of the bite force and its variation with opening angle from a reduced set of accessible parameters.

### 1.1. A biomechanical analysis of biting in arthropods

The magnitude of the bite force, |**F**_*b*_|, is determined by the architecture and the physiological properties of the musculoskeletal bite apparatus. These two components are fully captured by two simple terms: the net muscle force which pulls on the apodeme (from here on apodeme force); and the mechanical advantage of the force transmission system, *MA*, i.e. the ratio of two characteristic moment arms [53,56,85]. The net muscle force is the vector sum of the forces generated by individual muscle fibres. Combined, all fibres produce a force with a magnitude equal to the product between a characteristic muscle stress, *σ*, and a characteristic cross-sectional area, *A*_phys_. However, unless the fibres are perfectly aligned, only some fraction of the fibre force magnitude contributes to the magnitude of the net muscle or apodeme force. This fraction is typically characterized via an average angle of pennation, *ϕ* (throughout this article, all vectors are bold, and all unit vectors are indicated by a hat). We may thus write:

1.1 $$|F_{b}|(\theta )=\sigma (\theta )A_{\mathrm{phys}}\cos[\phi (\theta )]MA(\theta ).$$

**Figure 1.**
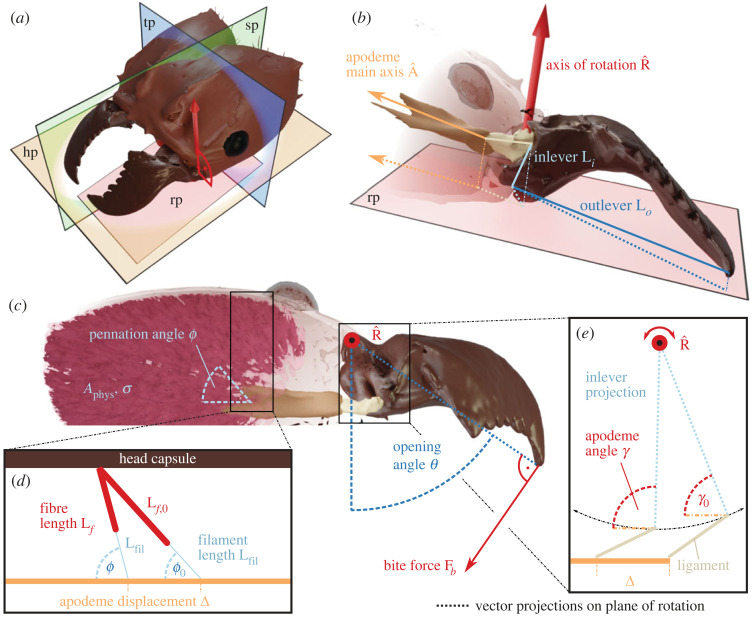
(*a*–*c*) The bite force **F**_*b*_ is a function of muscle stress *σ*, physiological cross-sectional area *A*_phys_, pennation angle *ϕ*, apodeme angle *γ* and the mechanical advantage determined by inlever **L**_*i*_ and outlever **L**_*o*_, the axis of rotation $\hat{R}$ and the apodeme main axis $\hat{A}$. The mechanical advantage and muscle stress, as well as the apodeme and pennation angle, vary with the mandibular opening angle *θ*, so affecting the magnitude of the maximum bite force that can be generated at different opening angles. *γ* and *θ* are defined with respect to the vector projections of $\hat{A}$, **L**_*i*_ and **L**_*o*_ onto the rotational plane (rp). The position of this plane with respect to the head coordinate system, defined by the horizontal (hp), transversal (tp) and sagittal (sp) plane, can often be inferred from joint morphology [85]. (*d*) Muscle fibres attach either directly or via thin filaments to the apodeme, the functional equivalent of a vertebrate tendon. When muscle fibres shorten (*L*_*f*,0_ to *L*_*f*_), the pennation angles increase from *ϕ*_0_ to *ϕ*, and the apodeme is displaced by Δ; the filament length remains approximately constant (see text). (*e*) As a result of the apodeme displacement, the inlever rotates about $\hat{R}$ and *γ* changes. Notably, the apodeme appears to move in pure translation, i.e. its main axis has a constant orientation. The lateral displacement of the attachment point which must accompany mandible rotation is likely accommodated by rotation of the putatively soft apodeme ligament, which connects the apodeme to the mandible base.

We note that only one of the four key parameters in this equation, *A*_phys_, is independent of the mandibular opening angle *θ*, defined here as the angle between the lateral head axis and the projection of the outlever onto the plane of rotation (figure 1*a*–*c* and [86]). In the following paragraphs, we derive the critical functions *σ*(*θ*), cos[*ϕ*(*θ*)] and *MA*(*θ*) from first principles. Understanding how the morphology and physiology of the bite force apparatus control the variation of bite force with the opening angle is not just an enjoyable exercise in mechanics, but has substantial biological implications, for it determines the accessibility of food items of different size [4], and potentially prey handling times [44]. However, this problem has received relatively little quantitative attention [4,10,44,84,86].

We derive bite force as a function of the opening angle because this relationship is biologically meaningful and reflects the typical experimental procedure to measure bite forces [22,26,60]. Physically, however, the bite force is not a function of the opening angle, but of the muscle activation and the muscle fibre stretch ratio *λ*. The causal relationship |**F**_*b*_| = *f*(*λ*) is provided in the electronic supplementary material. Throughout the derivation, we use the following assumptions: mandible kinematics are fully characterized by a single axis of rotation; the tendon-like apodeme connects to the mandible base via a single attachment point and displaces along its main morphological axis which coincides with the net muscle force vector; the head capsule, apodeme and mandible are rigid. Due to the abundance of dicondylic mandible joints across the insecta, which are putative hinge joints [87,88], and the high elastic modulus of sclerotized cuticle (Young’s modulus ≥1 GPa, [89,90]), these assumptions likely hold for the majority of all insect species with chewing-biting mouthparts, as well as for many other arthropods. The model thus has considerable generality, but we stress that it may not be readily applicable to vertebrate biting, which may involve multiple muscle insertion points, jaw articulations with multiple degrees of freedom, and more complex tissue deformations [91].

#### 1.1.1. Mechanical advantage

Musculoskeletal lever systems are often characterized in terms of their mechanical advantage: the ratio between two characteristic moment arms, *MA* = |**L**_*i*,eff_|/|**L**_*o*,eff_| [10,53,71]. These moment arms—the effective inlever, |**L**_*i*,eff_|, and the effective outlever, |**L**_*o*,eff_|—are determined by the location of two force transmission points, a reference point on the rotational axis, and the orientation of the apodeme force vector and the joint rotational axis (figure 1).

The points of force transmission are readily identified: *internally*, it is the attachment point of the apodeme to the mandible base (in most insects, muscle force is transmitted to the mandible via a single closer apodeme [88]); *externally*, it is the bite contact point on the mandibular blade. The rotational axis, $\hat{R}$, is determined by joint morphology and may often be inferred from the axis connecting the two joint condyles [10,61,62,86,92]. We use the apodeme main axis $\hat{A}$ as proxy for the orientation of the *internal* apodeme force vector because it is easier to measure and is likely closely aligned with the net muscle force vector [85]; where apodemes have multiple well-developed ‘branches’, a more suitable proxy may be derived from muscle geometry instead [10,86]. The *external* bite force vector **F**_*b*_, in turn, is perpendicular to both the rotational axis and the mandible outlever by definition (figure 1*c*).

Mathematically, the two moment arms are equal to the shortest distance between the *external* point of force transmission and the rotational axis (effective outlever), and the shortest distance between the rotational axis and the line of action of the apodeme force (effective inlever). To calculate both distances, we first define a specific plane of rotation by choosing a reference point on the rotational axis, in order to then find the planar (two-dimensional) components of the relevant three-dimensional position vectors (figure 1*a*). In theory, the choice of this point is arbitrary as it does not affect the moment arm calculation. In practice, however, it can be informed by joint morphology and may, in fact, be used to define the physical location of $\hat{R}$, e.g. by using the centre of a joint condyle. Secondly, we define the in- and outlever, **L**_*i*_ and **L**_*o*_, respectively, as the vectors connecting this joint centre with the internal and external points of force transmission. We project these vectors onto the plane of rotation and calculate the length of the projections, $\left|\hat{R}\times L_{i}\right|$ and $\left|\hat{R}\times L_{o}\right|$, respectively (figure 1*b*); these lengths are equal to the shortest distance between the axis of rotation and the two points of force transmission.

The lengths of the projected in- and outlever represent the maximum possible moment arms. The effective lever lengths, however, may be shorter, depending on their orientation relative to the relevant force vectors. Because the bite force is perpendicular to the outlever by definition, **F**_*b*_ rotates with **L**_*o*_ as the mandible opens and closes; it follows that $\left|L_{o,\mathrm{eff}}|=|\hat{R}\times L_{o}\right|$ for all opening angles.

For the inlever, in contrast, the geometric dependency is considerably more complex. The orientation of the apodeme force remains approximately constant across mandibular opening angles, because the apodeme does not rotate. However, because the joint converts apodeme translation into mandible rotation, the angle between inlever and $\hat{A}$ changes with opening angle (figure 1*c*,*e*). Calculating the magnitude of the effective inlever |**L**_*i*,eff_| at different opening angles thus requires to define the angle *γ* between the vector projections of inlever and apodeme main axis onto the plane of rotation. *γ* can be written as a direct function of opening angle, *γ*(*θ*) = *θ*_0_ − *θ* + *γ*_0_, where *γ*_0_ is measured at an arbitrary reference opening angle *θ*_0_ (for a similar calculation, see [10,84,86]). The effective inlever takes a maximum value of $\approx |\hat{R}\times L_{i}|$ for $\gamma \approx 90^{\circ }$; a small change in opening angle close to this maximum will be associated with a small change in effective inlever. If *γ* is acute or obtuse, however, the effective inlever is smaller than this maximum, and rapidly varies with opening angle. These results may be understood in analogy to the effect of pennation angle on apodeme displacement (see below): both angles control the amount of rotation associated with a unit of translational displacement. The two characteristic angles *ϕ* and *γ* thus crucially determine the gearing of the muscle force across opening angles. However, the importance of *γ* in this context has received comparatively little attention (but see [60]).

As a last step in the calculation of the mechanical advantage, we account for the fact that the apodeme force vector may not lie in the plane of rotation. The orientation of $\hat{A}$ relative to $\hat{R}$ matters because only the force components in the plane of rotation can be transmitted through the mandible joint; all other components result in joint reaction forces instead. We calculate the fraction of the force acting in the plane of rotation as the cross-product between the two relevant unit vectors, $\left|\hat{R}\times \hat{A}\right|$. The magnitude of the effective inlever as a function of the opening angle is expressed as follows:

1.2 $$|L_{i,\mathrm{eff}}(\theta )|=\sin(\theta_{0}-\theta +\gamma_{0})|\hat{R}\times L_{i}||\hat{R}\times \hat{A}|.$$

#### 1.1.2. Apodeme displacement

Any rotation of the mandible is associated with a displacement of the apodeme attachment point. This displacement has two components in the plane of rotation: one longitudinal component along the projection of the apodeme main axis and one lateral component perpendicular to it. Previous work has noted that the lateral component is small for small changes in opening angle and can thus be neglected [86]. This simplifying assumption, based on the small angle approximation, holds if the length of the inlever projection onto the plane of rotation is small, and the apodeme angle remains close to $90^{\circ }$ throughout the opening range. However, for large variations in opening angle, it is inaccurate. As an illustrative example, if *θ* changes by $40^{\circ }$, the lateral displacement is about one-third of the longitudinal displacement, $1/3\approx (1-\sin50^{\circ })/\cos50^{\circ }$ (figure 1*e*). The longitudinal displacement is likely associated with an equivalent displacement of the apodeme along its main axis; the lateral displacement, however, is likely not. If the apodeme was to translate laterally or rotate significantly, a fraction of the closer muscle would lengthen considerably, while another fraction may be slack. Indeed, the apodeme would eventually hit the head capsule. Lateral translation or rotation of the apodeme is thus biologically implausible, and to the best of our knowledge, has never been reported. However, lateral displacements of the attachment point must occur, because it moves along a circular path (figure 1*e*).

We hypothesize that these lateral displacements are enabled by the rotation of the apodeme ligament, a putatively flexible connective tissue which connects the sclerotized apodeme with the mandible (figure 1*e* [93–95]). It remains unclear how exactly the apodeme, ligament and mandible are mechanically linked. We conjecture that both longitudinal *and* transversal forces can be transmitted from the apodeme through the ligament, so effectively shifting the apodeme force vector to the point where the ligament attaches to the mandible. At this attachment point, we assume a hinge-like connection, which enables variation in *γ*.

Based on the this hypothesis, the displacement of the apodeme along its main axis, Δ, is equal to the longitudinal displacement of the apodeme attachment point corrected by the misalignment between $\hat{A}$ and the plane of rotation; in analogy to the effective inlever, only apodeme displacement components in the plane of rotation contribute to mandible rotation. Any out-of-plane displacements are likely also enabled by the ligament (and may typically be very small, see below). As a consequence and in analogy to equation (1.2), the apodeme displacement can be expressed as follows:

1.3 $$\Delta (\theta )=[\cos(\gamma_{0})-\cos(\theta_{0}-\theta +\gamma_{0})]\frac{\left|\hat{R}\times L_{i}\right|}{\left|\hat{R}\times \hat{A}\right|}.$$

We define Δ such that it increases as the mandible closes; however, it can be defined with respect to any reference opening angle *θ*_0_, for Δ(*θ*_0_) = 0. Similar to the effective inlever, the change of Δ with *θ* depends on the apodeme angle *γ*. If $\gamma \approx 90^{\circ }$, a small change in the opening angle will be associated with a large apodeme displacement; if *γ* is acute or obtuse, the opposite holds.

#### 1.1.3. Pennation angle

All muscle fibres attach to the rigid head capsule on one side, and insert onto the surface of the apodeme on the other. Consequently, muscle fibres that attach at a reference angle $\phi_{0}>0^{\circ }$ with respect to the apodeme main axis change their relative orientation when they shorten to move the apodeme—they rotate. The associated change in pennation angle *ϕ*, first derived by [96], depends on *ϕ*_0_, and the average distance between muscle fibre origins and insertion points, *L*_*t*,0_ (see figure 1*d* and electronic supplementary material for derivation):

1.4 $$\phi (\theta )=\arctan\left(\frac{\sin\phi_{0}}{\cos\phi_{0}-\Delta (\theta )/L_{t,0}}\right).$$

#### 1.1.4. Muscle stress

Muscle stress is a function of fibre length, as the relative overlap and lattice spacing between thick and thin myofilaments varies when fibres are stretched or contract [97–99]. In contrast to all other parts of the model, this force–length relationship cannot be easily derived from first principles (but see [100]). Instead, it has been modelled with a variety of empirical functions; one simple and thus attractive choice is a Gaussian probability density function [101,102]:

1.5 $$\sigma (\theta )=\sigma_{\max}\;e^{-\beta (1-L_{f}(\theta )/L_{\mathrm{opt}}{)}^{2}}.$$

This function has a maximum *σ*_max_ at an optimal fibre length *L*_*f*_ = *L*_opt_; *β* is a shape parameter which determines how quickly the stress decays as the fibre is stretched or contracts. Equation (1.5) contains three physiological parameters—*σ*_max_, *L*_opt_ and *β*—that cannot be easily measured from CT scans; they depend on the microanatomy of the muscle, such as the relative length of the myofilaments [98,103]. Note that *σ*_max_, *L*_opt_ and *β* may vary within the muscle between directly and filament-attached fibres [104], but the form of equation (1.5) holds for both. More complex functions of *σ*(*θ*), e.g. those which include passive muscle forces [101,105], may characterize the variation of muscle stress with opening angle more accurately, but at the cost of introducing an even larger number of parameters (see below).

#### 1.1.5. Fibre length

To predict muscle stress as a function of opening angle, the relationship between muscle fibre length and opening angle needs to be known. In insects, this relationship has previously been modelled with a simple linear function [10], which may be sufficiently accurate provided that the range of opening angles is small. For a large range of opening angles, however, the implicit use of the small angle approximation becomes inaccurate, and the exact functional relation is more complex. Consider a muscle fibre of total length *L*_*t*_ which attaches directly to the apodeme. The variation of fibre length *L*_*d*_ with apodeme displacement is determined by the reference fibre length *L*_*d*,0_, the fibre pennation angle *ϕ*_0_, and Δ(*θ*) (see [106] and electronic supplementary material for derivation):

1.6 $$L_{d}(\theta )=\sqrt{[\cos\phi_{0}L_{d,0}-\Delta (\theta ){]}^{2}+[\sin\phi_{0}L_{d,0}{]}^{2}}.$$

However, in some insects, fibres may attach via thin cuticular filaments instead [85,107]. Filament-attachment increases volume occupancy and cross-sectional area of the muscle while keeping the apodeme compact; it is a light-weight solution to strongly increase bite force capacity in the limited space provided by the rigid head capsule [85,108]. In addition to these benefits, filament-attachment also has implications for dynamics: consider two muscle fibres of equal length; one is directly attached and one filament-attached. The same fibre shortening will lead to a different apodeme displacement because filaments reduce the amount of rotation per unit fibre strain (figure 1*d*). As a consequence, the change in pennation angle associated with a unit fibre strain is smaller in the filament-attached fibre.

In analogy to equation (1.6), the relation between the length of filament-attached fibres and apodeme displacement depends on the initial fibre length *L*_*f*,0_, pennation angle *ϕ*_0_, Δ(*θ*) and filament length *L*_fil_ via (see electronic supplementary material for derivation):

1.7 $$L_{f}(\theta )=\sqrt{\begin{array}{cc} & [\cos\phi_{0}(L_{f,0}+L_{\mathrm{fil}})-\Delta (\theta ){]}^{2}\\ & +[\sin\phi_{0}(L_{f,0}+L_{\mathrm{fil}}){]}^{2} \end{array}}-L_{\mathrm{fil}},$$

where we assumed that filament length is independent of pennation angle (the strain in the filaments across the opening range is less than 2%, see electronic supplementary material).

The last parameter in equation (1.1) is the physiological cross-sectional area of the muscle, *A*_phys_. The physical cross-sectional area of the muscle changes as fibres change length, because muscle is approximately incompressible. *A*_phys_, however, is a characteristic cross-sectional area, and thus invariant to strain. In vertebrates, *A*_phys_ is often measured for a relaxed muscle (e.g. [109]), but this definition is problematic in comparative work, because the relaxed fibre length may be a different fraction of *L*_opt_ in different muscles. In order to avoid a systematic under- or overestimation of *σ*_max_, we thus suggest to define *A*_phys_ as the physiological cross-sectional area at an equivalent point of the force–length curve; the natural choice for this point is the cross-sectional area for fibres of length *L*_opt_ at which stress is maximal.

Based on the derived geometric relations, we substitute the place-holding functions in equation (1.1), *MA*(*θ*), *ϕ*(*θ*), and *σ*(*θ*), with equation (1.2) (divided by |**L**_*o*,eff_|), equation (1.4), and equation (1.5), respectively. Muscle stress is expressed as a function of fibre length, so that equation (1.6) (for directly-attached fibres) and equation (1.7) (for filament-attached fibres) need to be inserted into equation (1.5) as appropriate. Finally, fibre length and pennation angle are expressed as functions of the apodeme displacement, so that equation (1.3) needs to be inserted into equations (1.4), (1.6) and (1.7). The final equation contains a total of 20 physical parameters, including *θ*.

The derived model fully captures the determinants of bite force in arthropods and its variation with opening angle. Practically, the bite force is rarely measured directly. Instead, its magnitude, |**F**_*b*_|, is inferred from measurements with one- or two-dimensional force sensors [22,53,60,110]. As a consequence, at least one of its vector components has to be inferred from geometry. For one-dimensional sensors, the key geometric variable is the angle *α* between the force-sensitive axis, and the bite force, **F**_*b*_; the measured force, **F**_*m*_, is equal to the projection of **F**_*b*_ onto the sensitive axis:

1.8 $$|F_{b}|=\frac{\left|F_{m}\right|}{\cos\alpha }=\frac{|L_{o}\times \hat{R}||F_{m}{|}^{2}}{(L_{o}\times \hat{R})\cdot F_{m}},$$

where the term $L_{o}\times \hat{R}$ represents the orientation of **F**_*b*_. The inner product between $L_{o}\times \hat{R}$ and **F**_*m*_, divided by their respective magnitudes, is equal to cos *α*. The sensitive axis is determined by the sensor design; the orientation of **F**_*b*_, however, is subject to experimental variation, for it depends on the orientation of the mandible outlever relative to the axis of rotation (see above). The corresponding relationship between |**F**_*b*_| and |**F**_*m*_| for two-dimensional sensors is provided in the electronic supplementary material.

## 2. Material and methods

### 2.1. Study animals

To validate the derived geometric relations (equations (1.2)–(1.7)), we extracted the morphological determinants of bite force from insect heads of similar size, and prepared to represent a wide variation in mandibular opening angles. We then used the validated geometric model to extract the force–length properties of mandible closer muscles by conducting *in vivo* bite force measurements. For both sets of experiments, we use leaf-cutter ants (genus *Atta*). Leaf-cutter ants constitute an excellent model organism for at least three reasons. First, biting is a key to their ecology; they cut fragments from plant matter of varying thickness and toughness to feed a fungus grown as crop [111,112]. Second, the mandible closer muscle consists of both directly- and filament-attached muscle fibres [107], which enables us to verify the two separate geometric relations between fibre length and apodeme displacement (equations (1.6) and (1.7)). Third, mandibular opening angles observed during natural behaviour span a large range of around $70^{\circ }$ (see below), allowing us to validate the model across a maximum opening angle range.

Ants were collected from an *A. cephalotes* colony, kept in a climate chamber (FitoClima 12.000 PH, Aralab, Rio de Mouro, Portugal) at $25^{\circ }C$ and 60% relative humidity, with a 12/12 h light–dark cycle. The colony was fed with bramble, laurel, cornflakes and honey water ad libitum. We selected only majors with a body mass between 50 and 60 mg to minimize variation due to size, reduce the complexity of handling small individuals during the experiments, and to enable bite force measurements across a maximal opening range. All individuals were weighed to the nearest 0.1 mg after collection (Explorer Analytical EX124, max. 120 g × 0.1 mg, OHAUS Corp., Parsippany, NJ, USA).

### 2.2. Tomography and morphometric analysis for model validation

#### 2.2.1. Sample preparation and scanning

To obtain information on the morphological determinants of bite force, we conducted tomographic scans of five ant heads (body mass 55.0 ± 3.8 mg), prepared such that the mandibular opening angles approximately spanned the naturally observed range. To experimentally control the opening angle, live ants were clamped using a three-dimensional printed device and offered polylactic acid (PLA) rods of varying thickness to bite onto. The rods were positioned asymmetrically between the mandibles, so that the opening angles between left and right mandible differed (see electronic supplementary material for details). This procedure was followed for four of the five ants; one ant was prepared without PLA rod, so that mandibles were maximally closed. We thus collected data for 10 opening angles across the entire opening range. Ants were then sacrificed by freezing, causing muscles to forcefully contract, decapitated using micro scissors, and fixed in their contracted state in paraformaldehyde solution (4% in phosphate-buffered saline, Thermo Fisher Scientific, Waltham, MA, USA). After 90–95 h, the samples were transferred to 100% ethanol via a series of dehydration steps in 70, 80 and 90% ethanol solutions for an hour each. To prepare the heads for CT scanning, they were stained with 1% iodine in ethanol for at least 4.5 days [113]. The samples were analyzed via X-ray microscopy using a Zeiss Xradia Versa 520 X-ray microscope (Carl Zeiss XRM, Pleasanton, CA, USA; for more details, see electronic supplementary material).

The tomographic image stacks were reoriented such that the lateral, dorso-ventral and anterior–posterior head axes aligned with the coordinate system internal to Fiji ([85]; for more details, see [114]). Tissue segmentation of head capsule, mandible closer muscle and closer apodemes were performed in ITK-SNAP (v 3.6, [115]).

#### 2.2.2. Morphometry

From the segmented tomographic scans, a series of morphological parameters were extracted: the mechanical advantage, mandibular opening angle and the corresponding mandibular gape, the rotational axis of the mandible joint, apodeme orientation and displacement, volume of the mandible closer muscle, as well as average muscle fibre length and average pennation angle.

*Mechanical advantage*. The mechanical advantage was calculated as defined by equation (1.2). The relevant lengths, **L**_*i*_ and **L**_*o*_, and angles, *γ*_0_ and *θ*_0_, were extracted separately for each head hemisphere. The joint centre was placed at the ventral articulation of the joint (for an exact definition, see [85]). The outlever may be defined with respect to any point on the mandibular cutting edge; we used the most distal tooth tip to provide an upper bound, **L**_*o*_ = **L**_*o*,*d*_. We calculated the mandible gape as twice the shortest distance between the distal tooth tip and the sagittal plane of symmetry. The gape is thus negative when mandibles overlap.

*Axis of rotation*. To obtain the orientation of the rotational joint axis, $\hat{R}$, we invoke a simple result from rigid body kinematics: if the mandible rotates about a single axis, then the angle between any vector which connects two points on the mandible and $\hat{R}$ remains constant throughout mandible motion. $\hat{R}$ was thus determined by minimizing the squared angular residuals between $\hat{R}$ and three such vectors, **L**_*i*_, **L**_*o*,*d*_, and the outlever to the most proximal tooth tip, **L**_*o*,*p*_, using a numerical solver in python [116]. To integrate vector coordinates from different scans into the same coordinate system, the image origins were first transposed to the respective joint centres. Second, the coordinates were normalized with head length, defined as anterior–posterior distance between mandible joint and the rear of the head capsule. Finally, to combine data from the left and right head hemisphere, the coordinates of the latter were mirrored across the sagittal plane.

*Apodeme displacement*. To obtain the apodeme displacement, we first extracted the apodeme centres of mass via three-dimensional particle analysis in Fiji (for more details, see [85]). The centre-of-mass coordinates were transposed and normalized as described for the rotational axis to integrate results from different individuals into the same coordinate system. Next, we performed a principal component analysis on the normalized coordinates; the first component explained the dominant part of the variation of coordinates, *R*^2^ = 0.931, and thus coincides with the axis of displacement. We defined the apodeme displacement as the distance between the normalized coordinates along this axis, multiplied with the average head length. In support of the model assumption, the axis of displacement is close to the apodeme main axis $\hat{A}$, defined as in [85]; it differs only by $8\pm 2^{\circ }$ (mean±standard deviation), so that the net force is effectively aligned with $\hat{A}$ ($\cos(8^{\circ })\approx 1$). In the following, both axes are hence treated as equivalent.

*Muscle architecture*. The muscle volume was directly measured via ITK-SNAP. We have previously described an automated algorithm to extract fibre length and pennation angles from segmented tomography scans [85]. However, this tracing algorithm was developed for scans with a resolution and contrast only achievable with synchrotron scanning, to which we did not have access for this work. In addition, preliminary inspection of the scans revealed little ‘free volume’ between fibres, on which the automated tracing algorithm relies. We thus developed an alternative method based on a simple sorting algorithm. This algorithm leverages the observation that all muscle fibres originate from the internal surface of the head capsule and connect as approximately straight lines to a central apodeme either directly or via thin sclerotized filaments [85,107]. Obtaining pennation angle and fibre length with this algorithm involves three simple steps (see electronic supplementary material).

Firstly, the three-dimensional location of the fibre attachment points (seeds) on the head capsule are identified using Fiji as described previously (see [85] for details). Secondly, each fibre seed is connected to its insertion point on the apodeme surface. To identify the insertion point, we assume that the density of fibre attachments on the apodeme is approximately constant along its length and that muscle fibres rarely cross. On the basis of these assumptions, the fibre insertion point can be found by sorting fibre seeds and apodeme surface points by their anterior–posterior positions, to then match fibre seeds and apodeme surface points with the same relative rank. As a result, fibre seeds originating at the rear of the head are connected to posterior points on the apodeme, and seeds located closer to the mandible are connected to anterior points (see electronic supplementary material). To prevent fibres from crossing the apodeme, the apodeme surface point closest to the fibre seed was selected from all points with the same anterior–posterior rank. The orientation of the lines connecting fibre seeds to apodeme surface was then used to calculate fibre pennation angles with respect to the apodeme main axis. The length of the connection lines *L*_*t*_ was used to extract the muscle fibre length. For directly-attached fibres, *L*_*d*_ = *L*_*t*_. For filament-attached fibres, the fibre length was calculated as *L*_*f*_ = *L*_*t*_ − *L*_fil_, where *L*_fil_ is the filament length, obtained in a last step.

Thirdly, we extract filament length *L*_fil_ for each fibre. To this end, filaments are grown from their insertion points on the apodeme along the orientation of the respective fibre. After crossing at least 5 pixels of muscle tissue, approximately equal to the fibre diameter, the growth was terminated, and the filament length was extracted. Muscle fibres for which *L*_fil_ < 5 pixels were classified as directly-attached; fibres shorter than twice the fibre diameter were excluded from further analysis.

To quantify the quality of this ranking method, we manually extracted length and pennation angle of 200 fibres. The results of these direct manual measurements were compared with the results of the ranking method by matching the corresponding fibre seeds. On average, fibres from the ranking method were 1±18% shorter than those measured manually, independent of opening angle (linear mixed model (LMM) with random intercepts and opening angle as fixed effect: $\chi_{1}^{2}=0.51$, *p* = 0.48); the error of the pennation angle was small, $0\pm 1^{\circ }$. Although this error changed significantly with opening angle—pennation angles were increasingly underestimated at larger opening angles (LMM: $\chi_{1}^{2}=8.53$, *p* < 0.01)—it remained small even for large opening angles, $<3^{\circ }$. The quality of our simple sorting algorithm thus compares favourably to that of state-of-the-art commercial tracing software [109,117].

### 2.3. Bite force measurements

**Figure 2.**
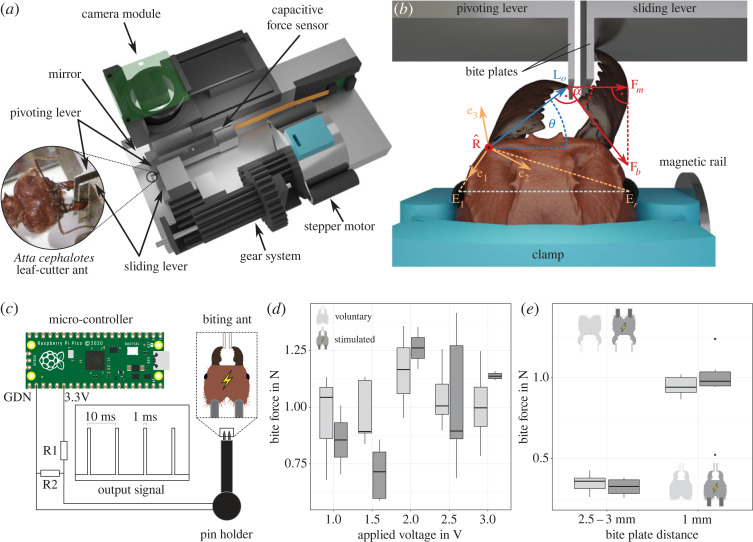
(*a*) A custom-built force rig was used to measure bite forces of *A. cephalotes* majors (photo by Victor Kang). In short, a force-sensitive capacitor was attached to a rigid metal frame. A pivoting lever presses onto it as a force is applied at the opposite end of the lever. A second sliding lever, controlled by a stepper motor, provides control over the distance between the bite plates. (*b*) To measure bite forces at small opening angles *θ*, ants were positioned off-centre using a three-dimensional printed clamp attached to a magnetic rail. Bite force |**F**_*b*_| was extracted from the measured force |**F**_*m*_| using the angle *α*, the outlever **L**_*o*_ and the joint axis of rotation $\hat{R}$, as defined in equation (1.8). $\hat{R}$ was projected onto a head-fixed coordinate system (**e**_1_, **e**_2_, **e**_3_), defined by the vectors connecting the mandible joint with left (**E**_*l*_) and right eye centres (**E**_*r*_). (*c*) A custom-built device was used to stimulate the mandible closer muscle, so eliciting maximum bite forces. A Raspberry Pi Pico was used to generate a square function as output signal with 100 Hz frequency and a duty factor of 0.1. The voltage was regulated using a voltage divider with varying resistors (0.08≤R1≤2.24 kΩ, R2=1 kΩ). A three-dimensional printed device was connected to the circuit. This device holds two insect pins, 1.5 mm apart and each protruding 2 mm. The pins were inserted into the head of live ants, positioned in front of the bite plates, and the device was activated. (*d*) Stimulated bite forces (dark grey) were compared against the maximum voluntary forces (light grey), recorded before stimulation for voltages between 1 and 3 V. For voltages ≥2 V, stimulated forces were approximately constant. For 1 V (shaded area), bites did not always occur; the ‘stimulated’ forces hence likely include voluntary bites. (*e*) Maximum voluntary and stimulated bite forces were of similar magnitude for both small and large opening angles (measured with a stimulation voltage of 2.75 V). Hence, ants bite with close to maximum available force independent of opening angle and undeterred by the rigid, metallic material of the levers, and the clamping.

To extract the force–length properties of the mandible closer muscle, we measured bite forces of *A. cephalotes* majors using a custom-built setup, described in detail in the study by Püffel *et al.* [118]. In brief, a capacitive force sensor (maximum load: 1 N, resolution: 0.002 N) is compressed when a force is applied to a bite plate connected to a pivoting lever (figure 2*a*). The position of a second slidable lever can be controlled by a stepper motor, so adjusting the distance between two bite plates. To extract mandible and head position during biting, a camera recorded the experiment simultaneously from top–down at 30 fps. A $45^{\circ }$-angled mirror provides a side view for a three-dimensional reconstruction of landmarks (see below). Force sensor, motor, and camera were controlled by a Raspberry Pi (v 3B+, Raspberry Pi Foundation, Cambridge, UK) and operated via a graphical user interface. Details on the sensor calibration are provided in the electronic supplementary material.

#### 2.3.1. Experimental protocol

Ant majors were collected from the foraging area or the fungus garden as available, and held in front of the bite plates of the force sensor using insect tweezers. Force recordings were terminated as soon as at least five distinct bites occurred. Subsequently, the ant was marked (Edding 4000 paint marker, Edding AG, Ahrensburg, Germany) and placed into a separate container with fresh bramble leaves and around 20 minors and medias to provide a resting period. After around 2 min, the same ant was measured again, but with a different plate distance. This process was repeated once more, so that bite forces of a single individual were measured three times at three different plate distances. Plate distances were chosen at random from three equally sized bins between 0.7 and 3.6 mm, approaching the maximum mandible gape range of the ants. To test if muscle fatigue may confound the results [119], an additional five ants were measured using the same protocol, but at a constant plate distance of 1 mm. A LMM with random intercepts revealed no significant effect of trial number on bite forces ($\chi_{1}^{2}=3.75$, *p* = 0.053; we note that this result is almost significant, but in the opposite direction than expected: bite forces were around 15% larger at the last trial compared to the initial measurement).

To comprehensively characterize the relationship between bite force and mandibular opening angle, it is necessary to measure bite forces across the largest possible range of opening angles. The upper end of this range can be approached by simply increasing the distance between the bite plates; the smallest angle, however, is structurally limited by bite plate thickness (approximately 150 μm each for our setup). To overcome this limit, we fixed the ant heads with a custom three-dimensionally printed clamp, magnetically connected to a metal frame. The clamp allowed us to position the ant heads off-centre relative to the bite plates, so enabling bites at lower opening angles (figure 2*b*). Clamped ants did bite much less frequently and sometimes only after stimulation with polite air blows (also see [60]). To test if clamping affected bite forces, five ants were clamped and positioned centrally in front of the sensor. The plate distance was selected at random to fall between 1.7 and 2.5 mm, and bite forces were measured. There was no significant difference in force between unclamped and clamped bites of the same gap class (two-sample t-test: *t*_28_ = 1.19, *p* = 0.24).

#### 2.3.2. Video analysis

We extracted the maximum force from each bite force trace (see electronic supplementary material for raw data examples). To avoid confounding effects due to variation in mandible outlever, only bites transmitted with the most distal tooth were considered. The following three-dimensional coordinates were extracted from the video frame corresponding to the peak bite force: the bite contact point (most distal tooth tip), joint centre, and the geometric centres of both eyes. The depth component of the three-dimensional coordinates was measured from the mirrored side view [118]. The vectors connecting mandible joint with both eyes **E**_*l*_ and **E**_*r*_ were used to define a local head-fixed coordinate system: **e**_1_ = **E**_*l*_/|**E**_*l*_|, **e**_3_ = (**E**_*l*_ × **E**_*r*_)/|**E**_*l*_ × **E**_*r*_| and **e**_2_ = **e**_3_ × **e**_1_. The rotational axis, $\hat{R}$, extracted from the tomographic scans, was projected onto this coordinate system using the same relative orientation to **e**_1_, **e**_2_ and **e**_3_ (figure 2*b*). The mandibular opening angle was calculated with respect to the lateral axis, here defined by the vector connecting both eye centres.

The capacitive force sensor has only one sensitive axis, i.e. it is a one-dimensional sensor. |**F**_*b*_| was hence extracted as defined by equation (1.8). Due to the design of the setup, an additional correction factor Γ was introduced to account for differences in moment arms due to variation in contact point on the bite plate. This correction factor reads $\Gamma =L_{\;p,\mathrm{cal}}/L_{\;p,b}$; *L*_*p*,cal_ is the moment arm used for calibration, and *L*_*p*,*b*_ is the moment arm defined by the mandible contact point on the bite plate.

#### 2.3.3. Electrical stimulation

To correctly interpret the results of the bite force measurements, we tested (i) if voluntary bites involve maximum muscle activation and (ii) if activation is independent of mandibular opening angle. We conducted these tests by eliciting maximum muscle activation through electrical stimulation of the mandible closer muscle.

To this end, a microcontroller (Raspberry Pi Pico, Raspberry Pi Foundation, Cambridge, UK; maximum output current 300 mA) was programmed to output a square function of stimulation impulses at 100 Hz frequency with a duty cycle of 10% (parameters used to elicit muscle contraction in moths [120] and beetles [121,122]). The output voltage was regulated using voltage dividers with resistors between 70 and 2230 Ω. A custom-designed device, consisting of two insect pins (size ‘0’, power and ground) and a three-dimensionally printed pin holder, was connected to the circuit (figure 2*c*).

Unclamped bite force experiments were performed following the aforementioned protocol with a fixed bite plate distance of 1 mm. This time, however, the measurement was not stopped after a sufficient number of bites. Instead, the pins were inserted from posterior into the head capsule of the biting ant such that each pin was approximately in the centre of the closer muscle. Subsequently, the stimulation was turned on for 1 s, and the resulting bite force was recorded. Due to the approximate positioning and size of the pins, we cannot exclude that the opener muscle was also stimulated. However, due to its small relative size in *Atta* ants (≈5% of the closer muscle in volume, see [85]), any fast twitch excitation co-contraction would only have minor effects on the net bite force.

The maximum force of distal bites during both natural and stimulated bites were extracted from each recorded force trace. Voltages between 1 and 3 V were used to find the minimum voltage at which the muscles were stimulated maximally, similar to the range used for beetle leg muscles [121]. At 1 V, bites were not always elicited; the stimulated maximum may thus often represent voluntary bites. At 1.5 V, the muscle may still have been sub-maximally stimulated, causing bite forces lower than for voluntary bites (figure 2*d*). A marked increase of stimulated bite force was visible at 2–2.5 V. For voltages ≥2 V, the average ratio between maximum voluntary and maximum elicited bite force remained constant (analysis of variance: *F*_1,9_ = 1.17, *p* = 0.31). On the basis of these results, we selected 2.75 V as excitation voltage for further stimulated bite force measurements at small (1 mm) and large (2.5–3 mm) plate distances. Occasionally, stimulated bites did not occur at the distal end of the mandible blade. In these cases, the calculated maximum bite force was corrected as |**F**_*b*,*s*_| = |**F**_*b*_||**L**_*o*,*c*,eff_|/|**L**_*o*,*d*,eff_| to account for differences in effective outlever, where **L**_*o*,*c*,eff_ and **L**_*o*,*d*,eff_ are the effective outlevers at the point of bite contact and the most distal tooth tip, respectively. We excluded stimulated measurements for which the difference in opening angle between unstimulated and stimulated bites exceeded $20^{\circ }$ to reduce confounding effects, and one additional measurement that exceeded the force range of the sensor calibration.

#### 2.3.4. Data analysis

The variation of morphological force determinants with mandibular opening angle can be directly predicted from the derived geometric relations (equations (1.2)–(1.7)). As reference parameters for these predictions, we selected the corresponding arithmetic average across all scans. To test for significant effects of opening angle on a variety of morphological parameters of the bite apparatus, we deployed linear mixed models: we tested if adding opening angle as a fixed parameter improves the model significantly compared to a random intercept model [123]. To extract the force–length properties of the muscle (equation (1.5)), maximum muscle stress, optimum fibre length and shape parameter *β* were estimated using a nonlinear least-squares fitting function in python, invoking equation (1.1). To enforce physical and biological plausibility, boundaries were set such that the maximum muscle stress and *β* are positive; the optimum fibre length was forced to remain within the range of measured fibre lengths. The physiological properties of directly and filament-attached fibres likely differ (see above and [104]); however, due to the small fraction of directly-attached fibres, 15±8%, we lacked sensitivity to determine their physiological properties with sufficient confidence. The physiological muscle parameters of these fibres were thus assumed to be equal to those of the filament-attached fibres, and *L*_opt_ was assumed to be at the equivalent stretch with respect to the average fibre lengths of both fibre populations. We merged data from all bite force experiments, as neither clamping nor measurement order affected bite force. We excluded the stimulated bites to avoid pseudo-replication and four further data points: three measurements from one individual that produced outliers at low opening angles ($55^{\circ }<\theta <65^{\circ }$; |**F**_*b*_| < μ − 3*σ*) and one measurement where the second mandible interfered during the experiment. Overall, we analyzed 138 force measurements from 81 individuals (54.8 ± 3.2 mg).

## 3. Results and discussion

Bite forces influence animal behaviour and relate directly to muscle physiology and head morphology. They have significant consequences for animal fitness and are thus under selection. As a consequence, bite forces may be considered a nonpareil performance metric for comparative studies at several levels of organismal biology [1]. We derived a detailed first principles model to describe the variation of bite force with mandibular opening angle across arthropods. To validate the geometric derivations of our model, we measured the relevant parameters from tomography scans of heads of *A. cephalotes* majors across a large mandibular opening range. We then used our model to estimate the physiological properties of the mandible closer muscle from *in vivo* bite force measurements via equation (1.1). In the next paragraphs, we (i) demonstrate the accuracy and predictive power of the model; (ii) discuss the magnitude and variation of leaf-cutter ant bite forces with opening angle in an ecological and comparative context; and (iii) present a ‘minimal’ model to predict bite forces from simple morphological measurements.

### 3.1. Variation of morphological bite force determinants with opening angle is in excellent agreement with model predictions

Our model derivation involved four key assumptions: firstly, we assumed that filament strain is negligible. This conjecture is supported by the observation that filament length is independent of opening angle (LMM: $\chi_{1}^{2}=0.32$, *p* = 0.58, see electronic supplementary material). Secondly, we assumed that the head capsule is effectively rigid. This assumption is supported by the fact that head dimensions vary little with opening angle (see electronic supplementary material). Thirdly, we conjectured the apodeme ligament rotates and so enables the lateral displacement imposed by mandible rotation. Indeed, we found evidence for such changes in the lateral expansion of the ligament (see electronic supplementary material). Fourthly, we modelled the mandible joint as a one degree-of-freedom hinge. The mandible joint in some ants, including leaf-cutter ants, has an unusual morphology [124], but mandible motion nevertheless approximates planar rotation across the large range of opening angles relevant to this study [116].

**Figure 3.**
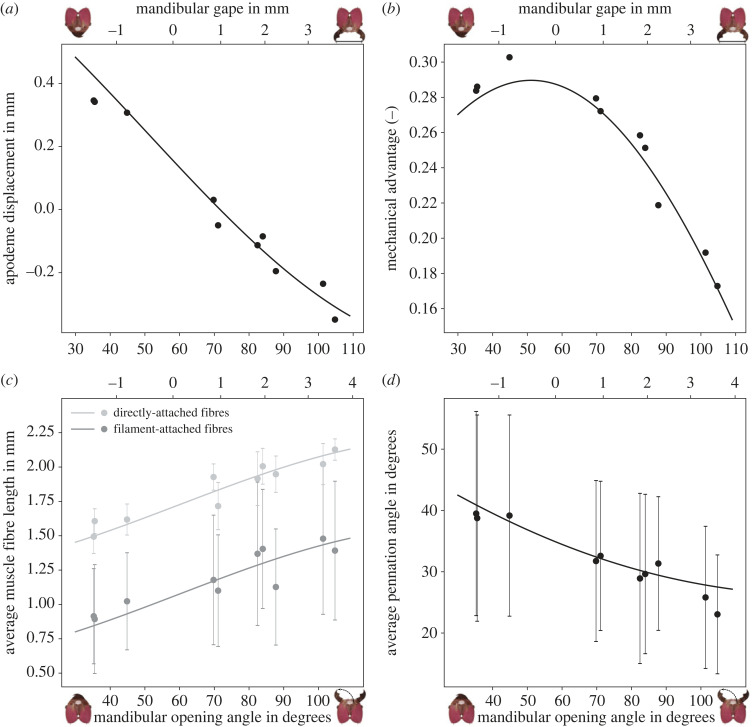
The variation of the relevant morphological parameters with mandibular opening angle, extracted from tomographic scans of *A. cephalotes* majors, is in excellent agreement with the theoretical predictions. Points and error bars represent the extracted mean±standard deviation. The solid lines show the model predictions based on the sample means (see text); they are not fitted lines. The mandible gape is shown on the upper abscissa. (*a*) Mandible closure is associated with a displacement of the apodeme. Across the opening range, this displacement amounts to around 0.7 mm, or about one-fifth of the average head length. Positive values represent displacements away from the average apodeme position in the posterior direction, and negative values indicate anterior displacements relative to the average position. (*b*) The mechanical advantage is maximal at $51^{\circ }$, close to zero mandible gape, and decreases by a factor of almost two at the largest opening angle. (*c*) The displacement of the apodeme is associated with a change in fibre length, which follows different relationships for directly and filament-attached fibres (equations (1.6) and (1.7)). The resulting *absolute* length changes are similar between both fibre populations (≈0.6 mm). However, as directly-attached fibres are much longer on average, their *relative* length change is much smaller. (*d*) As fibres shorten, they rotate and thus change their orientation. Across the opening range, the associated decrease in average pennation angle is around $13^{\circ }$.

Having provided empirical support for the simplifying assumptions involved in the model derivation, we turn our attention to the predictions it enables. Mechanical advantage, apodeme displacement, fibre pennation angle and fibre length are important morphological determinants of bite forces, which all change with mandibular opening angle (equations (1.2)–(1.7)). Direct morphological measurements of these parameters across a large range of opening angles are in excellent agreement with theoretical predictions based on arithmetic means, without exception (figure 3).

We measured opening angles between $35\;\mathrm{and}\;105^{\circ }$ for fully closed and maximally opened mandibles, respectively. At the maximum opening angle, the gape is 3.6 mm or approximately 75% of the head width. At the minimum gape, in turn, the most distal mandible teeth overlap by about 1.3 mm. To move the mandible from maximally opened to fully closed, the apodeme displaces by around 0.7 mm corresponding to about one-fifth of the average head length (figure 3*a*). This displacement may appear small, but it constitutes more than 50% of the average fibre length and thus requires large fibre strains (see below). At small opening angles, the apodeme angle *γ* is close to $90^{\circ }$ and varies approximately linearly with apodeme translation (this follows from the small angle approximation, $\cos(d\theta +90^{\circ })=-\sin(d\theta )\approx -d\theta$, equation (1.3)). At large opening angles, this linearity no longer holds, and the same change in opening angle is associated with a smaller longitudinal displacement of the apodeme attachment point, and a larger lateral displacement (figure 1*e*).

The mechanical advantage takes a maximum value of 0.29 at an opening angle of $51^{\circ }$, where $\gamma \approx 90^{\circ }$. As a consequence, at most 29% of the apodeme force is transmitted at the distal end of the mandible blade (figure 3*b*). The maximum *MA* is at the lower end of values reported for insects across numerous taxa (0.3 < *MA* < 0.8, see [88]). The *MA* decreases with the increasing opening angle in direct proportion to the effective inlever, and is reduced by a factor of 1.7 at the largest opening angle, at which *γ* has decreased to about $40^{\circ }$. The increasing steepness of this decline directly reflects the change in slope of sin(*γ*) (equation (1.2)). If the variation of *MA* was neglected, *MA*(*θ*) = *MA*_max_, the capacity for bite forces at large opening angles would thus be overestimated by about 70%. This variation is similar to the range of possible effective outlevers from the most distal to the most proximal sections of the mandibular blade for our study organism and across various insect taxa [88]. The fact that animals can control *MA* by changing the outlever has previously been discussed in an evolutionary context [88]; that a similar effect, which alters bite force just as much across the opening range, arises from variations in opening angle, however, has received very little attention in both the vertebrate and invertebrate literature (but see [84,86]).

The displacement of the apodeme is associated with a change in muscle fibre length. Both directly and filament-attached fibres shorten by around 0.60 mm across the opening range, starting from a similar maximum *total* length of 2.11 and 2.07 mm, respectively (figure 3*c*). The absolute changes in the fibre length are similar, but with an average filament length of 0.60 mm—equivalent to around 75% of the smallest fibre length—the relative changes in fibre length differ substantially. The ratio between largest and smallest fibre length is 1.41 for directly-attached fibres, but 1.73 for filament-attached fibres, considerably larger than ratios reported for other insects (1.35 and 1.55 for the mandible closer muscle in stag beetles and cockroaches, respectively, [10,86], and 1.50 for the extensor tibiae muscle in stick insects [125]). Such large changes in relative muscle length are associated with a substantial decrease in muscle stress [97,98], which seemingly favours a direct muscle attachment over filament attachment. The abundance of directly-attached fibres, however, is likely limited by space constraints in the head capsule; filament-attachment increases the effective internal muscle attachment area to the apodeme, so that its net effect may still be an increase in muscle force [85,107].

In addition to changes in the fibre length, the apodeme displacement is associated with changes in average pennation angle (figure 3*d*). *ϕ* is around $41^{\circ }$ at low opening angles and decreases to $27^{\circ }$ at large angles. The notable standard deviation of the measured angles (and fibre lengths, figure 3*c*) does not reflect measurement error (see method validation), but the multi-pennate architecture of the closer muscle in *Atta* ants [85,104]. The decrease of *ϕ* is steepest at small opening angles and flattens towards larger angles (figure 3*d*). This pattern is driven by two effects: firstly, at large opening angles, the apodeme attachment point displaces mostly laterally due to the small apodeme angle. As a consequence, the longitudinal displacement, which controls fibre rotation, is small. Secondly, the change in pennation angle per unit apodeme displacement is determined by the magnitude of the pennation angle itself and is largest for large pennation angles.

The decrease in *ϕ* increases the fraction of the muscle force aligned with the apodeme by about 20%, $\cos(27^{\circ })/\cos(41^{\circ })\approx 1.18$, more than twice as much as reported for cockroaches [86]. This increase is nevertheless small and insufficient to balance the reduction in force due to the decrease in *MA* (figure 3*b*). There exists a further notable difference between the two parameters: the pennation angle will always be maximal for small opening angles and then decrease monotonously to a minimum at large opening angles (figure 1). In sharp contrast, the effective inlever is largest at $\gamma =90^{\circ }$, which could be achieved at any opening angle by relatively small changes in mandible shape, so resulting in non-monotonous variation across the opening angle range. These observations suggest two different functional roles for the pennation and apodeme angle: the variation in pennation angle is likely irrelevant for the change in force magnitude across opening angles. However, large pennation angles increase the maximum possible physiological cross-sectional area in the limited volume of the rigid head capsule, and thus control the magnitude of the bite force [53,85,107]. Pennation also results in ‘displacement gearing’ [126,127], which reduces the fibre strain required to cover the same range of opening angles. This reduction is important because muscle stress may decay steeply with fibre length; it is, however, difficult to draw direct conclusions from it because the interactions among pennation angle, fibre strain, muscle stress and the maximum possible muscle size in a fixed volume are subtle, and require a more detailed parametric analysis than is within the scope of this study. The apodeme angle, in turn, does not meaningfully alter the maximum magnitude of the bite force. However, because it can increase or decrease with the opening angle, it can be used to counter or magnify any changes in bite force arising from force–length effects—it is a more flexible gearing parameter across the opening range than the pennation angle. The possible ‘design space’ span by these two parameters is thus large and presents an interesting area for comparative work.

We have demonstrated that the variation of the key morphological design parameters of the insect bite apparatus with opening angle can be accurately predicted from first principles. Next, we show that knowledge of these relationships can be used to extract the physiological properties of insect muscle, which remain understudied (but see [104,125]).

### 3.2. Leaf-cutter ants are extremely specialized for large bite forces, and bite strongest at opening angles relevant for cutting

We used the deductive power of our geometrically validated biomechanical model to extract the force–length properties of the mandible closer muscle. To this end, we performed *in vivo* bite force measurements across a large range of opening angles, $50-105^{\circ }$, and extracted the magnitude of the maximum bite force at each opening angle via equation (1.8). To test if the ants bit with maximum activation, we compared voluntary and electrically stimulated bite forces. There was no significant difference between the maximum voluntary and stimulated bite forces for either small or large bite plate distances (Welch two-sample t-test, 1 mm distance: *t*_5.68_ = −0.04, *p* = 0.97; 2.5–3 mm distance: *t*_9.69_ = −1.76, *p* = 0.11, figure 2*e*). Consequently, ants appear to maximally activate their muscle during our bite force measurements, independent of the mandibular opening angle, and undeterred by the rigid material of the bite plates (but see [128]).

To characterize the physiology of the mandible closer muscle, we extracted maximum muscle stress, optimum fibre length, and the force–length shape parameter *β*, via a nonlinear least-squares fit of equation (1.1). The resulting fit yielded optimum fibre lengths of *L*_opt_ = 1.42 mm (95% confidence interval (CI) (1.17; 1.66)) for directly-attached fibres, and *L*_opt_ = 0.92 mm (95% CI (0.79; 1.07)) for filament-attached fibres. We used *L*_opt_ to extract the physiological cross-sectional area of the muscle at a defined point on the force–length curve (see above), *A*_phys_ = *V*_*m*_/*L*_opt_ = 4.3 ± 0.4 mm^2^. The maximum muscle stress was fitted as 1.16 MPa (95% CI (1.06; 1.26)), at the upper end of values reported for arthropod muscle, which are typically below 1 MPa ([10,53,60,62,125,129], but see [16,23]). We caution against strong conclusions on the basis of this comparison, because most of the published muscle stress estimates do not represent the true maximum muscle stress, as they do not stem from direct or indirect measurements of force–length properties. Based on the difference in average sarcomere length, it is likely that the true stress of some crab pincher muscles, for example, exceeds our estimate for leaf-cutter ant closer muscle [23,104], which may suffer further from shrinkage effects arising from CT sample preparation [130].

The shape parameter was fitted as *β* = 5.34 (95% CI (0.09; 10.59)). Data on the force–length properties of arthropod muscle are surprisingly scarce. To assess the plausibility of our result, we extracted force–length data from published work, normalized all forces with maximum force, all lengths with optimum length, and fitted equation (1.5) with a nonlinear least-squares algorithm. The shape parameter for *Atta* closer muscles is close to the shape parameter for fibres from the extensor carpopoditi muscle of the European crayfish (*β* = 6.27, 95% CI (5.72; 6.84) [131]), the extensor tibiae muscle of Indian stick insects (*β* = 3.95, 95% CI (3.29; 4.71) [125]), fibres from the meropodite muscle of walking legs of Orconectes crayfish (*β* = 6.61, 95% CI (5.87; 7.41) [132]) and the abdominal ventral superficial muscle of Hermit crabs (*β* = 6.46, 95% CI (5.25; 7.87) [133]). However, it is substantially smaller than values for extensor muscles in the coxa of discoid cockroaches (*β* = 12.18, 95% CI (11.10; 13.30) [134]), the respiratory muscle in green crabs (*β* = 26.66 , 95% CI (13.07; 56.23) [135]) and the (synchronous) mesothoracic dorso-longitudinal muscle of *Manduca sexta* moths (*β* = 58.65, 95% CI (52.89; 64.59) [136]). In light of the considerable variation in both sarcomere lengths and the ratio between thick and thin filaments in arthropod muscle [23,137], this large range may not be surprising and suggests exciting opportunities for comparative muscle physiology.

**Figure 4.**
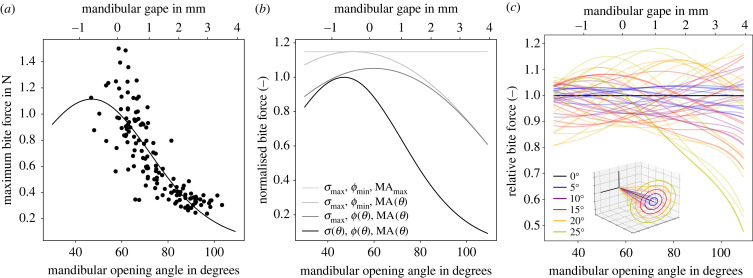
(*a*) Bite forces of *A. cephalotes* majors were measured across mandibular opening angles between 50 and $105^{\circ }$. Bite forces peak at small opening angles and decrease to around one-fifth of that maximum at $100^{\circ }$. The solid line is a least-squares fit of equation (1.1), which accounts for the variation of both morphological and physiological determinants of bite force. For the largest opening angles, the model underestimates the measured forces, which may be partially attributed to passive joint and muscle forces (see electronic supplementary material). The peak muscle force associated with peak bite forces is 9000 times larger than the mean body weight of the ants—a factor of seven larger than expected for animals of this size (see text). (*b*) The variation in bite force is driven by changes in muscle stress and mechanical advantage. The pennation angle, on the other hand, mainly modulates the maximum bite force magnitude. The black line represent the fitted relationship between bite force magnitude and opening angle, |**F**_*b*_|(*θ*), normalized with its maximum. The grey lines show the theoretical relationships between bite force and opening angle, if muscle stress, the cosine of the pennation angle, and the mechanical advantage were progressively fixed at constant values equal to their respective maxima. If these parameters were all maximal at the same opening angle, the maximum bite force would increase by a mere 15%. Because muscle volume occupancy is also close to its maximal theoretical value [85], leaf-cutter ant heads are close to a morphological optimum for maximum bite forces. (*c*) The variation of bite force with opening angle strongly depends on the mandible joint axis of rotation. A seemingly small variation in this axis of only $15^{\circ }$ changes the relative bite force prediction by up to 30%. The coloured lines represent the theoretical relationships between bite force and opening angle for 50 randomly generated axes of varying angles to the actual axis (black), normalized with the fitted relationship, |**F**_*b*_|(*θ*). Clearly, the axis of rotation is a crucial parameter if the variation of bite force with opening angle is to be predicted accurately.

Our biomechanical model captures the general shape of the variation of bite force with opening angle both quantitatively and qualitatively. The agreement between prediction and observation, however, is arguably less convincing than for the geometric relations alone in at least two aspects (figure 4*a*). Firstly, at the largest opening angles, the fit systematically underestimates the measured bite forces, which remain approximately constant for $\theta >90^{\circ }$. This disagreement partially reflects passive joint or muscle forces, which may contribute about 50 mN (see electronic supplementary material [60,105]). Second, close to the fitted maximum, the spread of measured bite forces is large. At small opening angles, the misalignment angle between measurable and bite force vector is most extreme (equation (1.8)). The increased variation may thus represent a combination of biological variation and small errors in landmark placement, amplified by larger correction factors. For measurements at small opening angles ($<65^{\circ }$), the average correction was 28±25%, compared to only 5±4% for measurements at larger angles.

We measured maximum bite forces of 1.4 N, equivalent to about 2600 times of the mean body weight of the ant majors. Maximum bite forces were measured at small opening angles ($<65^{\circ }$, figure 4*a*), and bite force decreased steeply with opening angle to a minimum of 0.3 N at $100^{\circ }$—a total variation of a factor of five. Even considering the comparatively small size of the ants (50–60 mg), these weight-specific forces are rather remarkable indeed: Alexander conducted a literature review of the maximum forces exerted during various activities, including biting, by animals spanning ten orders of magnitude in mass [83]. He reported an upper bound for weight-specific forces of *F*_max_ = 20 m^−1/3^, where *m* is the body mass in kg. The expected value for a 55 mg leaf-cutter ant is *F*_max_ = 20 (55 10^−6^)^−1/3^ ≈ 500. Leaf-cutter ants thus bite with a force around five times larger than expected for an animal of their size; these values even surpass those from other unusually strong animals such as crabs [23]. The specialization of leaf-cutter ants to produce large bite forces is even more startling in the context of muscle force: leaf-cutter ants exert maximum muscle forces of *σ*_*m*_ *A*_phys_ ≈ 5 N—around 9000 times their body weight. An approximate upper bound for maximum weight-specific muscle force is 50 m^−1/3^ [83], or ≈1300 for leaf-cutter ant majors—almost an order of magnitude smaller than the observed value. Clearly, leaf-cutter ants are extremely specialized to produce large bite forces.

Having fully characterized peak bite forces, and the key physiological and morphological parameters of the mandible closer muscle in *Atta* majors, we can assess the relative importance of physiology or morphology for the bite force–gape relationship (equation (1.1)). The majority of this variation is driven by changes in muscle stress, which varies by a factor of 5.7 (figure 4*b*); the mechanical advantage decreases by a factor of 1.7. Both effects are slightly attenuated by the decrease in pennation angle, which effectively increases the bite force for large opening angle by 18%. Remarkably, if all parameters were optimum at the same opening angle, the maximum bite force would increase by only ≈ 15%. Compared to the significant variation in muscle stress and mechanical advantage, this ‘lost’ potential appears rather small, and it is mainly driven by the pennation angle, which is anatomically constrained (see above). The bite apparatus in leaf-cutter ants thus seems to have an architecture close to a theoretical optimum; the muscle volume density approaches its theoretical maximum [85], and pennation angle, apodeme angle, and optimum fibre length are all ‘synchronized’ such that bite force peaks in a narrow range of opening angles (centred around $47^{\circ }$). The opening angle range where bite forces are maximal corresponds to small mandible gapes (<0.5 mm). This range is most critical in the context of the typical behaviour displayed during leaf-cutting, because fresh cuts are often initiated with ‘scissor-like’ cuts through the leaf lamina, and the average lamina thickness of tropical leaves is around 0.25 mm [138]. When propagating cuts through the lamina, ants often use a large range of opening angles [139]; however, when encountering thick veins that require high bite forces, mandibles open only very little (see electronic supplementary material in [140]). Leaf-cutter ants may thus adjust their cutting behaviour in accordance with the steep decrease of bite force for opening angles $>75^{\circ }$. These results represent a key step towards understanding the intricate relationship between cutting behaviour, maximum bite force and mandibular opening angle in *Atta* foraging (also see [118]).

### 3.3. Minimal models for the magnitude of bite force and its variation with gape

We have demonstrated that both the magnitude of bite force and its variation with gape can be predicted from first principles. Many individual components of our biomechanical model have been discussed by colleagues previously [10,53,56,60,84,85,141–143]. However, a comprehensive analysis which combines all elements into one single model has, to our knowledge, been absent from the literature. The advantage of a complete first principle model is that it is as accurate as the estimates of the relevant parameters which define it. The problem is that many of these parameters are time consuming and costly to measure. Accordingly, there has been considerable interest in ‘minimal’ bite force models, where some parameters are either replaced with first-order approximations, scaling relationships or proxies identified via statistical analysis [1,3,26,28,56,141,143–146]. As an illustrative example, head width has repeatedly been shown to correlate tightly with bite force in vertebrates, which, combined with conserved maximum muscle stress, seemingly makes bite force predictions rather straight forward [6,28,143]. Due the extraordinary attractiveness of a minimal bite force model for ecologists, palaeontologists, evolutionary biologists and biomechanists alike, we next discuss the implications of our analysis for such models in insects; firstly, with respect to the magnitude of the absolute bite force and then for its variation with gape. In probing the accuracy with which the magnitude of the maximum bite force can be predicted without full knowledge of all parameters, we are assessing an upper bound, corresponding to the assumption that all relevant parameters take their maximum value at the same opening angle (figure 4*b*). By separating the question of bite force variation with gape from its absolute value, we are then considering only *relative* changes of bite force with gape.

The maximum distal mechanical advantage of the mandible bite apparatus in insects varies between 0.3 and 0.8 across a broad range of insect orders [88]. Using an intermediate value of *MA* ≈ 0.55 is thus associated with an uncertainty of less than a factor of two. A more accurate proxy may be obtained by measuring the ratio between mandible width and length [53]. The mandible length is likely a relatively accurate proxy for the outlever because the uncertainty associated with the location of the joint centre will be small compared to its length. The mandible width, however, may be a poor proxy for the inlever, which is typically shorter than the outlever, but subject to the same absolute uncertainty.

Average pennation angles of mandible closer muscles in insects are smaller than $45^{\circ }$ (data exist for ants, beetles and cockroaches [85,86,107,147]). The direct influence of pennation on bite force magnitude is thus negligible, cos *ϕ* ≈ 1.

An accurate measure of *A*_phys_ is perhaps most challenging to obtain: it requires knowledge of muscle volume, and crucially, the optimal fibre length. *L*_opt_ is typically unknown and can only be estimated from transmission electron microscopy images of muscle tissue (to measure thick and thin filament length and lattice spacing), or via knowledge of all other parameters and bite force measurements (this study). Using the physical cross-sectional area of muscle at an arbitrary but naturally occurring muscle length is associated with an error directly related to the strain range of the muscle: because muscle is incompressible, any change in its length is associated with a change in its cross-sectional area such that volume is conserved, *A* = *V*/*L*. If *L*_opt_ was overestimated by 70%, corresponding to the maximum fibre length ratio extracted in this study, *A*_phys_ would be underestimated by 1−1/1.7≈40%. However, even this proxy requires knowledge of muscle volume and fibre length, and thus CT data and advanced fibre tracing algorithms [85]. Due to these difficulties, a large number of proxies have been used in the literature, including head size (see below), the surface area of the apodeme [14] or the muscle attachment area to the head capsule [10]. Head size is arguably the most attractive proxy for *A*_phys_, for it represent the best compromise between accuracy and ease of measure; head volume can be readily estimated from head length, width and height, *V*_*h*_ ≈ *H*_*w*_ *H*_*h*_ *H*_*l*_, with simple light microscopy. In insects, both mandible closer muscles typically occupy between 10 and 50% of the total head volume *V*_*h*_ ([85,86,147,148], but see [108]). The volume of a single closer muscle can thus be estimated as *V*_*m*_ ≈ 0.3/2 *H*_*w*_ *H*_*h*_ *H*_*l*_. *L*_opt_, in turn, is some fraction of a linear head dimension. The most suitable dimension may be head height, as the region where it takes its maximum value is mainly occupied by closer muscle [85,86,147,148]). It follows that *L*_opt_ ≈ 0.5 *H*_*h*_, and *A*_phys_ = *V*_*m*_/*L*_opt_ ≈ 0.3 *H*_*w*_ *H*_*l*_. This estimate has an error similar to the suggested approximation for the mechanical advantage and is as good as the implicit yet untested assumption of isometry across animals of wildly different sizes.

The maximum muscle stress is the parameter most prone to variation; estimates for arthropods vary by almost two orders of magnitude (0.04– 2.2 MPa [16,23,60,125,129]). This large range limits the accuracy of a mean stress to one order of magnitude at best [60], and is likely at least partially owed to the experimental methods with which stress has been estimated. Force measurements with isolated insect muscle let alone single fibres are rare (e.g. [125,129,134]). Most often, stress is estimated from more integrated bite/pinch force measurements instead (e.g. [23,53,60] and this study). Any such estimation of muscle stress, however, is subject to the uncertainties in determining *MA* and *A*_phys_ discussed above. A more accurate peak muscle stress estimate may be obtained from the average fibre sarcomere length, *S*_*l*_ ([23], but see [47]). To this end, we extracted all non-vertebrate data from the classic work of Taylor and conducted a standardized major axis regression, *σ* ∼ *S*_*l*_, in log-log space. For sacromere lengths between 3 and 17 μm, this relationship reads *σ*_max_ = 50 *S*_*l*_, where *S*_*l*_ is in μm and *σ*_max_ is in kPa, and explains about 75% of the variation in stress with sarcomere length. The remaining variation is likely attributed to variations of the relative fibre length at which forces were measured and generic measurement error. The scaling coefficient of unity is robust (and has a theoretical foundation), but the proportionality constant of 50 kPa μm^−1^ is again subject to uncertainty in *A*_phys_, required to convert the measured force to a stress (e.g. our measurements in *Atta* are a noteworthy outlier). Taylor estimated *A*_phys_ as the surface area of the apodeme multiplied with sin *ϕ* ([23] also see [15,53]). The error in measuring apodeme surface area is likely small, but the error in assuming that it is equal to the muscle attachment area is ≥10% (the maximum packing density of a lattice of circles). The total uncertainty associated with this approximation for stress is thus at least 35%. Although measuring sarcomere length is technically involved, the significant reduction in uncertainty for the maximum stress associated with it may make these measurements well worthwhile.

We conclude the discussion for maximum bite force by summarizing that the simplest reasonable estimate for the maximum bite force requires only knowledge of head width and length. However, this estimate may at worst only be accurate to less than an order of magnitude. The next best estimation replaces the maximum stress with a proxy based on sarcomere length, and reads:

3.1 $$F_{\max}=\underset{0.55}{\underbrace{MA}}\;\underset{1}{\underbrace{\cos\phi }}\underset{0.3H_{w}H_{l}}{\underbrace{A_{\mathrm{phys}}}}\underset{50S_{l}}{\underbrace{\sigma_{max}(S_{l})}}\approx 8.25\;\text{kPa}\;\mu {\text{m}}^{-1}H_{w}H_{l}S_{l}.$$

If we use an intermediate sarcomere length of 10 μm [23], equation (3.1) simplifies to *F*_max_ ≈ 83 kPa *H*_*w*_*H*_*l*_. On average, this upper bound is approximately three times larger than values measured for 653 insect species (assuming $H_{w}H_{l}=V_{h}^{0.67}$, see [63]). Notably, these force measurements were conducted at arbitrary opening angles which may differ from the angle of maximum force. As demonstrated earlier, large deviations from this angle may lead to substantially lower bite forces (factor of up to five). For 12% of the measured species, our prediction underestimates the maximum bite force, which likely reflects species-specific adaptations. Overall, the ratio between maximum predicted and measured bite force ranges from 0.30 to 27, across almost two orders of magnitude. The effort required to measure sarcomere length is likely rewarded with a reduction in such worst-case errors by at least a factor of five.

Next, we address the variation of bite force with gape, which has received even less attention than maximum bite force, but is of similar ecological significance [26,84]. The cosine of the average pennation angle will typically vary at most between unity and 0.7 between maximally open and fully closed mandibles, respectively ($\cos0^{\circ }$ and $\cos45^{\circ }$, see earlier); this variation is small enough that it can be neglected, cos[*ϕ*(*θ*)] ≈ constant. Instead, the variation of bite force with opening angle is likely dominated by changes in muscle stress due to changes in muscle length; such effects are particularly pronounced when fibre strain and shape parameter *β* are large, and *L*_opt_ occurs at fully open or closed mandibles (this study). *β* appears to cluster around ≈6, but may be twice as large in some muscle (see above). The variation of the mechanical advantage can be significant, but much of this variation is governed by the apodeme angle *γ*, which can be measured relatively easily. Perhaps the most important *and* most neglected source of variation comes from the joint rotational axis. $\hat{R}$ affects virtually all bite force determinants, apart from *A*_phys_, and also influences the accuracy of bite force measurements themselves see equation (1.8)). In order to gain some intuition for both the error and the variation of force–gape curves which can be associated with variations in $\hat{R}$, we calculated the relationship between |**F**_*b*_| and opening angle using a set of 50 alternative rotational axes, located at the same joint centre, but oriented at an angle between 5 and $25^{\circ }$ relative to the original $\hat{R}$ (all other parameters were kept constant). The resulting variation, normalized with the original relationship |**F**_*b*_|(*θ*), is substantial, and includes a large variety of force-gape relationships (figure 4*c*). As illustrative examples, an axis deviating by only $10^{\circ }$ alters the force prediction by up to 20% compared to the original relationship |**F**_*b*_|(*θ*); an axis deviating by $25^{\circ }$ causes changes of up to 50%. Misidentifying the joint centre would lead to additional significant variation affecting both levers, the predicted apodeme displacement, and all associated parameters. For species with two well-developed condyles, the location and orientation of $\hat{R}$ may be identified with reasonable accuracy from joint morphology alone, as previously done for beetles [10,147], cockroaches [86] and dragonflies [61]. Other insects such as some hymenopterans, however, have more complex mandible joints [149,150], which may also have more than one degree of freedom [93,151,152], rendering such deductions difficult (but see [116,124]). Given the sensitivity of bite forces to the joint axis, it is surprising how little attention has been paid to rigorously determine mandible joint kinematics across the insect tree of life using the tools of rigid body mechanics (but see [116,151]). Any attempt to predict or measure the bite force–gape relationship needs to take due note of the importance of $\hat{R}$ to avoid significant errors.

## 4. Conclusion and outlook

We presented a validated model which predicts the bite force–gape relationship in arthropods from first principles, based on a minimum set of assumptions. We hope that our work will be useful for future studies on the bite performance in arthropods in at least three aspects. Firstly, it enables in vivo extraction of force–length properties from bite force measurements, which remain scarcely reported in the literature. The significant variation of the physiological make-up of insect muscle suggests exciting avenues for comparative muscle physiologists, and our model allows colleagues to side-step challenging measurements with tiny isolated muscle fibres. Secondly, our model identifies the key determinants of maximal bite forces, including a ‘minimal’ bite force model, and so facilitates comparative analyses of the functional morphology of the insect bite apparatus, including rare, small or extinct species, for which bite force measurements may be impossible. Third, it provides clear guidance on how muscle morphology and physiology translate into a variation of bite force with gape, which is of significant ecological and behavioural relevance, e.g. in the analysis of feeding guilds or niche formation. Indeed, the increasing number of high-resolution CT scans of insect heads offers tantalizing opportunities to study the bite force–gape relationship across the insect tree of life without the need for direct bite force measurements. Notably, systems that operate close to the maximum possible bite force, such as in leaf-cutter ants, will suffer from a steeper decrease in bite force at opening angles departing from the optimum, suggesting that the functional morphology of the bite apparatus is subject to a trade-off. All three points will contribute to our general understanding of the performance, behaviour, ecology and evolution of arthropods.

## Data Availability

We provide all model derivations in the electronic supplementary material [153].
